# Characteristics of Mesenchymal Stem Cells Originating from the Bilateral Inferior Turbinate in Humans with Nasal Septal Deviation

**DOI:** 10.1371/journal.pone.0100219

**Published:** 2014-06-13

**Authors:** Se Hwan Hwang, Sun Hwa Park, Jin Choi, Dong Chang Lee, Jeong Hoon Oh, Sung Won Kim, Jin Bae Kim

**Affiliations:** 1 Department of Otolaryngology-Head and Neck Surgery, The Catholic University of Korea, College of Medicine, Seoul, Korea; 2 Division of Gastroenterology, Department of Internal Medicine, Kangnam Sacred Heart Hospital, Hallym University, Korea; Rutgers - New Jersey Medical School, United States of America

## Abstract

**Background and objectives:**

Nasal septal deviation (NSD) is often associated with overgrowth of the unilateral inferior turbinate. *In vivo* and *in vitro* studies indicate that human mesenchymal stem cells (MSCs) are able to differentiate into multiple cell types, including osteoblasts. We tested the hypothesis that turbinate size affects human turbinate-derived MSC (hTMSCs) quantity, proliferation, and differentiation into osteogenic lineages, and that hypertrophic turbinates may predispose to NSD on the contralateral side.

**Subjects and Methods:**

The hypertrophic and contralateral inferior turbinate tissues used in our study were obtained and cultured from the tissue discarded from 10 patients who underwent septoplasty and partial turbinectomy. After isolating the hTMSCs from both turbinates, the cells were enumerated using an automated cell counter. The expression of surface markers for MSCs over four passages was assessed by fluorescent-activated cell sorting analysis (FACS), and cell proliferation was assessed using a cell counting kit (CCK)-8 according to turbinate size. In addition, osteogenic differentiation of hTMSCs was identified using alkaline phosphatase (ALP) and alizarin red S staining, after which osteoblastic gene expression was evaluated.

**Results:**

There was no significant difference in the number of hTMSCs. FACS analysis revealed that the hTMSCs were negative for CD14, CD19, CD34, and HLA-DR, and positive for CD29, CD73, and CD90, representing a characteristic MSC phenotype, with no significant difference between the two groups. The cellular proliferation and osteogenic differentiation potential of the hTMSCs were also not significantly different between the two groups.

**Conclusions:**

We conclude that turbinate size does not affect the characterization, proliferation, and osteogenic differentiation potential of hTMSCs in vitro test, and therefore should not affect the clinical decision of whether to use autologous or allogenic hTMSCs. However, more experiments are required to definitively state the relationship of hTMSCs with turbinate size or the process NSD in humans.

## Introduction

Nasal septal deviation (NSD), a convexity of the septum from the midline, is the most common deformity of the nose [1]. NSD towards one side is often associated with an overgrowth of the inferior turbinate, which occupies the expansive space of the contralateral nasal cavity [2]. It has been assumed that this counterbalanced mechanism, characterized by compensatory mucosal hypertrophy, serves to protect the more patent nasal side from excess airflow, which causes drying and crusting. However, recent radiological and histopathological studies have indicated that turbinate bone hypertrophy is primarily responsible for inferior turbinate hypertrophy (ITH) in patients with NSD and that mucosal hypertrophy is less important [3], [4].

Mesenchymal stem cells (MSCs) are a heterogeneous population of stem/progenitor cells with pluripotent capacity to differentiate into mesodermal and non-mesodermal cell lineages, including osteocytes, adipocytes, chondrocytes, myocytes, cardiomyocytes, fibroblasts, myofibroblasts, epithelial cells, and neurons. MSCs reside primarily in the bone marrow, but also exist in other sites such as adipose tissue, peripheral blood, cord blood, turbinate tissues, and maxillary sinus mucosa [5], [6]. In addition, we identified MSCs isolated from human inferior turbinate tissue (hTMSC) in a previous study [7]. Considering that the ITH with NSD showed significant bone growth rather than mucosal hypertrophy, it is possible that a patient’s inferior turbinates could be raised unequally, leading to continuous pressure on the nasal septum and causing NSD to the contralateral cavity. Also, if two inferior turbinates contain an unequal number of MSCs, or the hTMSCs undergo proliferation or osteogenesis, the two turbinates may develop asymmetrically.

We hypothesized that cell counts or proliferation potential would be higher in hTMSCs isolated from the hypertrophic turbinate than the contralateral turbinate and the existence of a turbinate-size-related increase in the osteogenic potential of hTMSCs. In this study, we aimed to validate the hypothesis that turbinate size affects hTMSC count, proliferation, and differentiation into osteogenic cell lines.

## Materials and Methods

### Ethics Statement

All studies using hTMSCs were performed after written approval (KC08TISS0341) from the institutional review board of Seoul St. Mary’s Hospital, the Catholic University of Korea and after obtaining written informed consent from the donors themselves. Investigations were conducted according to the principles expressed in the Declaration of Helsinki. Inferior turbinate tissues were discarded from 10 patients undergoing septoplasty and partial turbinectomy. The patients with history of facial trauma were excluded.

### Cell Isolation and Counting

An identical amount (0.0366 g) of bilateral inferior turbinate tissues (the mucosa of the hypertrophic turbinate in the broad nasal cavity and contralateral turbinate in the narrow nasal cavity) was obtained from discarded tissue during surgery and washed three to five times with saline containing gentamicin (Kukje Pharmaceutical Industries, Sungnam, Korea). To isolate the hTMSCs, the inferior turbinate tissue was washed three times at room temperature with antibiotic–antimycotic solution (Gibco, Gaithersburg, MD), twice with phosphate-buffered saline (PBS), and then cut into 1-mm^3^ pieces. The pieces were placed into a culture dish that was covered with a sterilized glass cover slide. Dulbecco’s modified Eagle’s medium (DMEM; Gibco) containing 10% fetal bovine serum (FBS) was added, and the tissues were incubated at 37°C in 5% CO_2_; the culture medium was changed every 2 or 3 days. After 3 weeks of culture, the glass cover slide was removed, and tissues floating in the culture medium were removed by washing. The hTMSCs that had attached to the bottom of the culture dish were detached using 3 ml of 0.25% trypsin in 1 mM EDTA; cells were then enumerated using an ADAM automated cell counter (Digital Bio, Seoul, Korea) to identify any significant difference between the hTMSCs from the bilateral inferior turbinate tissues.[7] The hTMSCs after four passages were examined for turbinate size-related changes in immunophenotypic characteristics, proliferation, and osteogenic differentiation.

### Immunolabeling and Flow Cytometry of Surface Markers on hTMSCs

Flow cytometry using the hTMSCs was conducted for the cell-surface markers CD14, CD19, CD29, CD34, CD73, CD90, and HLA-DR. The hTMSCs were placed into a test tube (BD, Franklin Lakes, NJ, USA) at 1×10^5^/ml and washed three times with wash buffer (PBS and 3% FBS). The cells were incubated for 40 min with saturating concentrations of the primary monoclonal antibodies against CD14, CD19, CD29, CD34, CD73, CD90, and HLA-DR. After the cells were washed three times in buffer and centrifuged at 1200 rpm for 5 min, they were resuspended in ice-cold PBS and incubated with a FITC- or PE-labeled secondary antibody for 30 min in darkness at 40°C. Cell fluorescence was evaluated using flow cytometry with a FACS Caliber instrument (BD); the data were analyzed using the Cell Quest software (BD).

### Proliferation of hTMSCs

The growth curves of hTMSCs from the hypertrophic inferior turbinate and the contralateral normal turbinate were determined over a period of 7 days. The cells were simultaneously plated into a 96-well tissue culture plate at 1500 cells per well. The media were replaced every 2 days. Cellular proliferation was assessed using a cell counting kit (CCK)-8 (Dojindo Laboratories, Kumamoto, Japan), per the manufacturer’s instructions.

### Evaluation of Osteogenic Differentiation of hTMSCs

To induce osteogenic differentiation, hTMSCs isolated from turbinate tissue were seeded in 12-well tissue culture plates (2×10^4^/well) and incubated in low-glucose DMEM supplemented with 10% FBS, 100 U/ml penicillin, 100 µg/ml streptomycin, 0.1 µM dexamethasone, 50 µM ascorbate-2-phosphate, and 10 mM β-glycerophosphate (all from Sigma).

### Real-time Quantification of mRNA

Total RNA was extracted from the cells cultured for osteogenic differentiation using TRIzol reagent (Invitrogen). The RNA was extracted as recommended by the manufacturer (Kontes, Vineland, NJ, USA). For cDNA synthesis, total RNA (2 µg) from each sponge was diluted to a volume of 10 µl in DEPC/DDW and used in a reaction (10 µl) containing 4 µl of 5×first-strand buffer (Invitrogen), 4 µl of DTT (0.1 M, Invitrogen), 1 µl of dNTPs (10 mM, Sigma), 0.5 µg of oligo (dT) 15 primer (Invitrogen), 1 µl of RNase inhibitor (Invitrogen), and 200 units of SuperScript II (Invitrogen). The reaction was incubated at 45°C for 60 min. The cDNA was stored at −20°C until needed.

The PCR reactions (50 µl) contained GoTaq Flexi DNA polymerase (Promega, Madison, WI, USA), 4 µl of MgCl_2_ (25 mM, Invitrogen), 2 µl of dNTPs (10 mM, Sigma), cDNA template, and primers specific for one of the following: bone sialoprotein (BSP), Runt-related transcription factor 2 (Runx2), bone morphogenetic protein-2 (BMP-2), osterix (OSX), osteocalcin (OC), and collagen type I (Col1) (Table 1). The thermal conditions for PCR amplification were: initial denaturation at 94°C for 3 min, followed by 35 cycles of 94°C for 30 s, 53°C for 30 s, and 72°C for 30 s, with a final extension at 72°C for 5 min. PCR products were separated in 2% agarose (Sigma) gels in 1× Tris-acetate-EDTA buffer containing ethidium bromide.

**Table 1:** Oligonucleotide primer sequences used for reverse transcriptase polymerase chain reaction (RT-PCR).

Gene	Primer	Sequence
BSP	forward primer (FP)	5′-GCT CAG CAT TTT GGG AAT GGC-3′
	reverse primer (RP)	5′-CTG CAT TGG CTC CAG TGA CAC-3′
Runx2	forward primer (FP)	5′-GTG GAC GAG GCA AGA GTT TCA-3′
	reverse primer (RP)	5′-TGG CAG GTA GGT GTG GTA GTG-3′
BMP2	forward primer (FP)	5′-TTG CGG CTG CTC AGC ATG TT-3′
	reverse primer (RP)	5′-CAT CTT GCA TCT GTT CTC GGA A-3′
Osx	forward primer (FP)	5′-CTT CAG TCT TCC CAA CTT CTT ACA C-3′
	reverse primer (RP)	5′-ACA AAT TGG GTT AGC TAC ATC TCT G-3′
OC	forward primer (FP)	5′-ATG AGA GCC CTC ACA CTC CTC-3′
	reverse primer (RP)	5′-CGG GCC GTA GAA GCG CCG ATA-3′
COL1	forward primer (FP)	5′-TGA CGA GAC CAA GAA CTG-3′
	reverse primer (RP)	5′-CCA TCC AAA CCA CTG AAA CC-3′

### Statistical Analyses

Statistical analyses were conducted using the statistical software SPSS (SPSS Inc., Chicago, IL, USA). The characteristics of the isolated hTMSCs from 10 patients, such as cell density, proliferation and osteogenic differentiation were divided into two groups according to turbinate size. Statistical comparisons between two groups according to turbinate size were performed using a *t*-test. A value of *p*<0.05 was considered to indicate statistical significance.

## Results

### Cell Count and Surface Marker Expression

**Figure 1:**
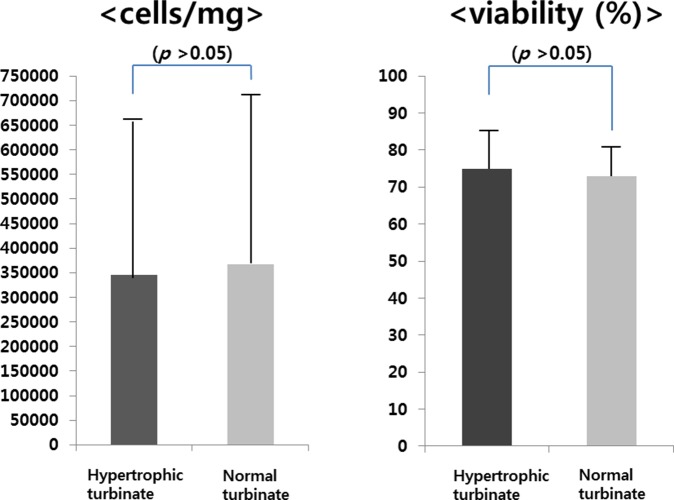
Turbinate size-related changes in the number and viability of hTMSCs. After isolation from the inferior turbinate, hTMSCs were enumerated using an automated cell counter. There was no significant difference in the number of cells between the hypertrophic and contralateral turbinates.

**Figure 2:**
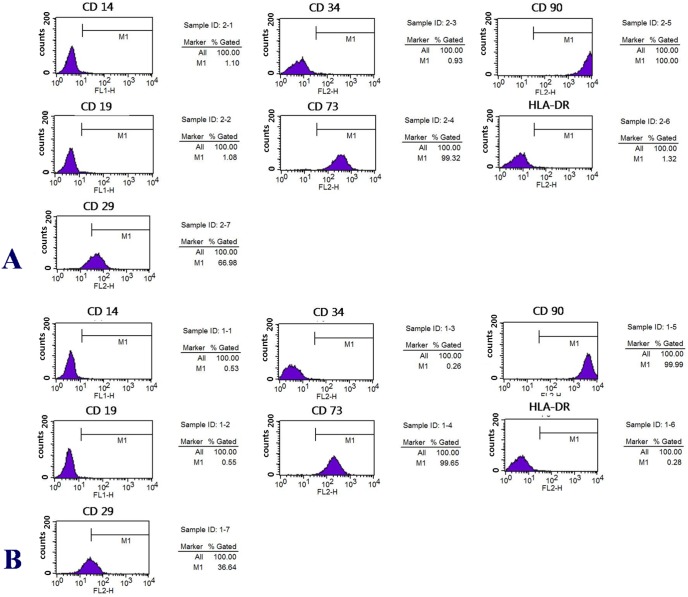
Turbinate size-related changes in immunophenotypic characteristics. hTMSCs in the hypertrophic turbinate (A) and the contralateral turbinate (B) were negative for CD14, CD19, CD34, and HLA-DR, and positive for CD29, CD73, CD90, a phenotype characteristic of mesenchymal stem cells. The hTMSCs in the two groups expressed a comparable proportion of specific surface markers.

The mean numbers of hTMSCs from the hypertrophic and contralateral turbinates were 3.46×10^5^±3.21×10^5^ and 3.66×10^5^±3.67×10^5^, respectively. There was no significant difference between the groups (Figure 1;Table 2). FACS analysis revealed that hTMSCs were negative for CD14, CD19, CD34, and HLA-DR, and positive for CD29, CD73, and CD90, representing a characteristic phenotype of mesenchymal stem cells; there was no significant difference between the groups (Figure 2).

**Table 2:** Data of patients with nasal septal deviation showing their cellular count and viabilty of hTMSCs.

Patients	Hypertrophic turbinate	Normal turbinate
	Cell counts per milligram (viability %)	Cell counts per milligram (viability %)
1	145000 (60%)	95000 (61%)
2	66000 (74%)	73000 (64%)
3	93000 (89%)	192000 (76%)
4	68300 (85%)	79300 (84%)
5	482000 (50%)	966000 (69%)
6	765000 (69%)	563000 (80%)
7	71900 (82%)	49290 (66%)
8	199000 (76%)	79600 (69%)
9	744000 (85%)	695000 (86%)
10	819000 (83%)	873000 (78%)

### Proliferation Assay

**Figure 3:**
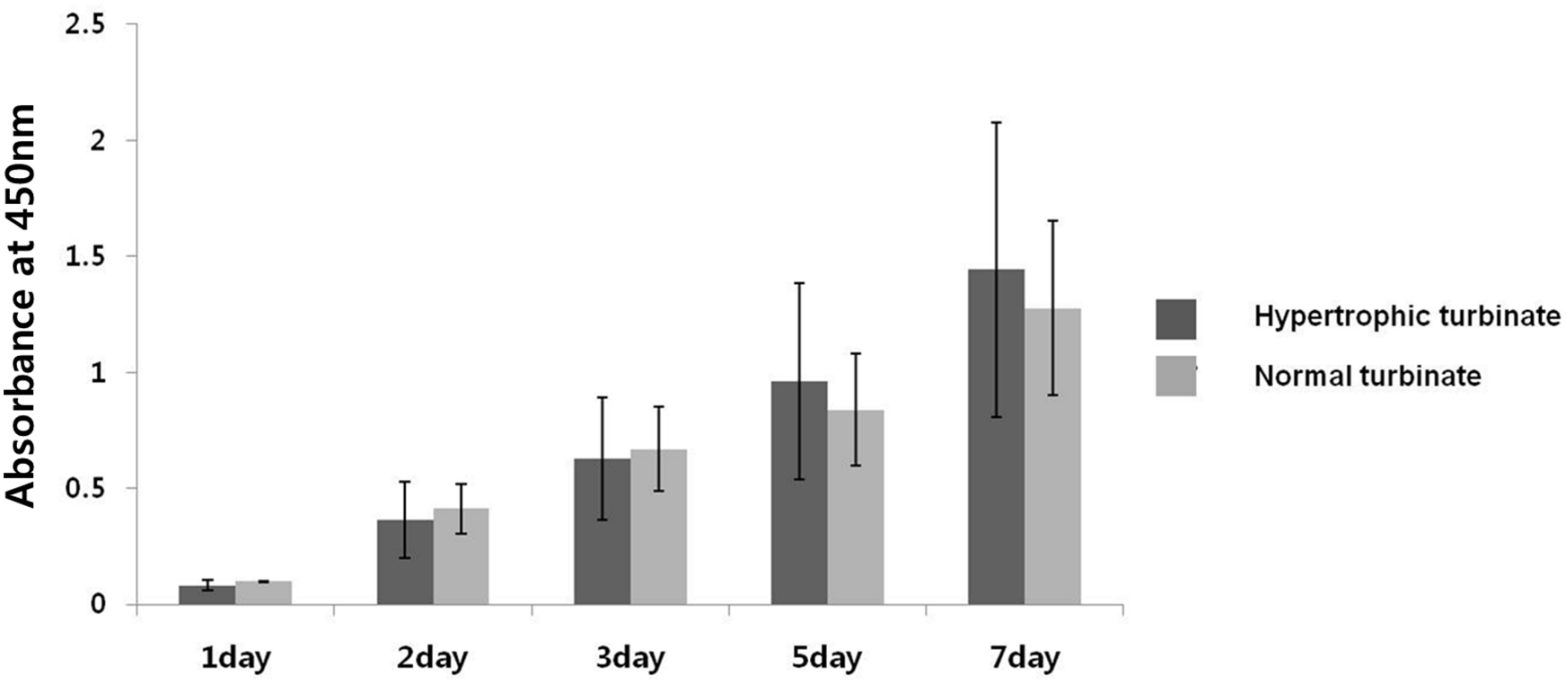
Effects of turbinate size on hTMSCs proliferation. Cellular proliferation was monitored for 7 days. hTMSCs from a hypertrophied inferior turbinate exhibited less proliferation than those from a normal-sized inferior turbinate from days 1 to 3, while hTMSCs from the hypertrophied inferior turbinate expanded more rapidly compared to those from the normal-sized inferior turbinate from days 5 to 7. There was a significant difference in proliferation between the two groups from days 5 to 7 (*p*<0.05). However, the hTMSCs from both groups exhibited a similar proliferation rate during the period of culture.

Proliferation of hTMSCs from the two groups according to turbinate size was assayed over 7 days. The hTMSCs from the hypertrophied inferior turbinate exhibited less proliferation than those from the contralateral turbinate from days 1 to 3, while the hTMSCs from the hypertrophied inferior turbinate expanded more rapidly than those from the contralateral inferior turbinate from days 5 to 7. The difference in proliferation between the two groups from days 5 to day 7 was statistically significant (Figure 3).

### Osteogenic Differentiation Potential

**Figure 4:**
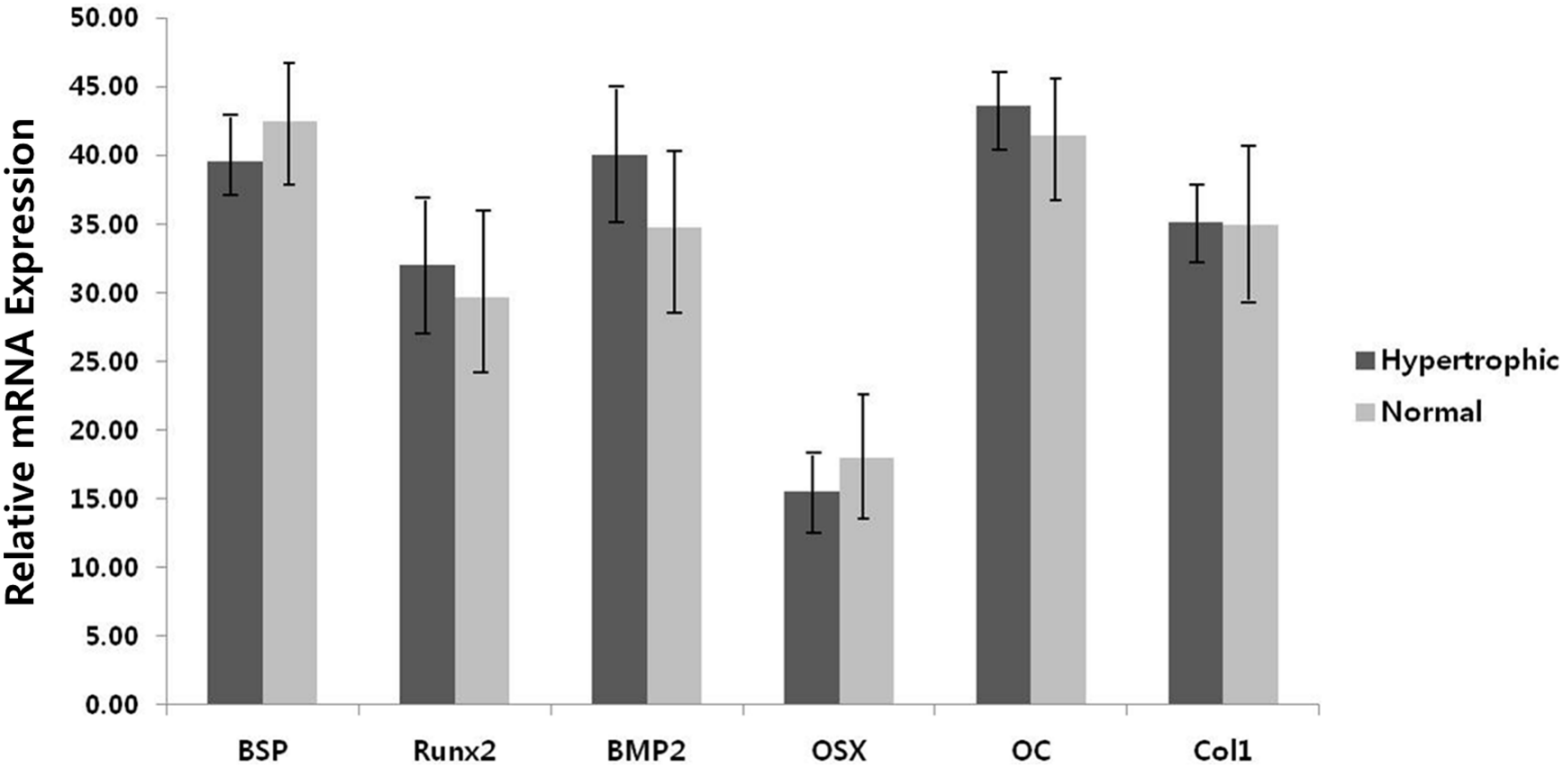
Effect of turbinate size on the osteogenic differentiation potential of hTMSCs. Cells were cultured in osteogenic induction medium. RT-PCR analysis of bone sialoprotein (BSP), runt-related transcription factor 2 (Runx2), bone morphogenetic protein-2 (BMP-2), osterix (Osx), osteocalcin (OC), and type I collagen (Col1) mRNA in osteogenically differentiated hTMSCs during 2 weeks of culture. The experiment was repeated in triplicate for each sample. No significant difference between the groups was identified by unpaired t-test (*p*<0.05).

RT-PCR analysis revealed the presence of mRNAs for osteoblast-specific genes encoding BSP, Runx2, BMP-2, OSX, OC, and Col1 in osteogenically induced hTMSCs over 14 days. Quantitative analysis of the expression of osteoblast-specific genes revealed no difference between the groups (Figure 4).

## Discussion

NSD, or a convexity of the septum from the midline, has an overall prevalence of 22.38% in Koreans; males tend to be overrepresented as are those of increased age [8]. NSD constitutes one of several causes of nasal obstruction or mouth breathing, prompting many patients to visit otolaryngology departments. Although the development of NSD is assumed to be determined by genetic, cultural and environmental factors, the etiology behind the condition is not yet understood [9].

The turbinates exist as three, and sometimes four, bilateral extensions from the lateral wall of the nasal cavity. Of the three turbinates, the inferior turbinate is most susceptible to hypertrophy. ITH can be bilateral or unilateral, and the bilateral variety can be due to hypertrophy from allergic or nonallergic rhinitis [2]. Enlargement of the erectile mucosa of the inferior turbinate significantly increases nasal airway resistance, contributing greatly to symptoms of nasal airway obstruction [10]. In contrast, unilateral enlargement occurs in association with a congenital or acquired anatomical deviation of the septum into the contralateral nasal passage. In patients with compensatory ITH secondary to NSD, the main cause of ITH is the bone, whereas the contribution of the medial mucosa is insignificant. It should be remembered that various mechanisms are implicated in prolonged nasal obstruction originating from marked bilateral ITH and in compensatory ITH [11]. Considering the above mentioned findings, it is likely that genetically determined primary unilateral growth of the turbinate bone exerts pressure on the growing nasal septum during childhood and adolescence, eventually causing it to bend toward the contralateral side of the nose [2].

Stem cells have the capacity for extensive self-renewal and for originating at least one type of highly differentiated descendant. Post-natal tissues have reservoirs of specific stem cells that contribute to maintenance and regeneration. MSCs, which reside in virtually all post-natal organs and tissues, act as a reservoir of undifferentiated cells to supply the cellular (and non-cellular) demands of the tissue to which they belong, acquiring local phenotypic characteristics [12]. When necessary, in response to environmental cues, they give rise to committed progenitors that gradually integrate into the tissue [5]. In our previous studies, we found that fibroblasts isolated from the inferior turbinate tissue discarded during turbinate surgery were multipotent mesenchymal stromal cells, which we refer to as human turbinate mesenchymal stromal cells (hTMSCs); these showed excellent potential for differentiation of osteoblasts from chondrocytes [7], [13]–[15].

Currently, no studies have revealed the cause of overgrowth of the unilateral inferior turbinate associated with NSD. In this study, we focused on the functions of the MSCs in the maintenance and regeneration of the tissues to reveal the mechanism of the asymmetric growth of bilateral inferior turbinates. Recent findings suggest that a decline in the numbers, proliferation, or potential of stem cell populations in adult organs may contribute to characteristics of human aging, such as the decline in bone mass and age-related diseases including osteoarthritis and osteoporosis [16], [17]. In addition, although it has not been reported that a greater number of mesenchymal stem cells in the tissue increases its volume, Troken et al.[18] suggested that higher mesenchymal stem cell densities yielded more marked matrix synthesis *in vivo* implantation. Mineral apposition is not attenuated by seeding hMSC-derived osteoblasts at a high density or in close proximity to each other.

In the present study, we compared the characteristics of hTMSCs from hypertrophied and contralateral normal inferior turbinate tissues obtained from 10 patients. We evaluated their distribution by cell counting and FACS, and the proliferation and osteo-differentiation of the hTMSCs were assayed. Cells from the hypertrophic and contralateral turbinates were cultured to isolate hMSCs individually, and cells were counted separately. There were no significant differences in the cell count and viability of the hTMSCs in the hypertrophic and contralateral turbinates. In FACS analysis, hTMSCs from both turbinates exhibited a phenotype characteristic of mesenchymal stem cells, and there was no significant difference between the turbinates. These results indicate that the distribution of MSCs in the hypertrophic and contralateral turbinates may not be related to turbinate size.

In the 7-day cellular proliferation assay, hTMSCs from the hypertrophied inferior turbinate exhibited less proliferation than those from the normal-sized inferior turbinate from days 1 to 3, while hTMSCs from the hypertrophied inferior turbinate expanded more rapidly than those from the normal-sized inferior turbinate from days 5 to day 7. There was a significant difference in proliferation between the two groups from days 5 to 7. However, the proliferation pattern of hTMSCs was similar between the hypertrophic and normal turbinate groups. These findings suggest that turbinate size did not significantly affect the proliferation of hTMSCs.

The three distinct phases of MSC differentiation and bone formation comprise proliferation, extracellular matrix maturation and matrix mineralization [19]. The first stage of cell proliferation occurs within the first 4 days. Early cell differentiation occurs during the second stage, which spans days 5 to 14, and is characterized by the transcription and protein expression of Col1 and alkaline phosphatase (ALP). Terminal differentiation and matrix maturation occurs during the third stage, from day 15 to day 28, which results in high expression of OP, BSP, and OC, followed by calcium and phosphate deposition [20]. Runx2 and Osx are primary osteoblast-specific transcription factors for osteoblastic differentiation [21], which positively regulate OC and BSP expression [22]. BMP-2 is known to regulate the mechanism upstream of Runx2 in osteogenic differentiation [23]–[25]. After culturing in osteogenic media, the expression levels of osteoblast-related genes were determined by RT-PCR. The expression levels of the other osteoblast-associated genes were not different between the two groups. These findings suggest that turbinate size does not significantly affect the osteogenic capacity of hTMSCs.

This study was the first to analyze the etiology of ITH secondary to septal deviation based on MSCs rather than histological and radiological findings. In particular, the cause of turbinate bone hypertrophy had not been evaluated. Based on the previous findings that MSCs reside in virtually all post-natal organs and contribute to their maintenance and regeneration, and because turbinate size does not affect the characteristics, proliferation, and osteogenic differentiation potential of hTMSCs, the hypertrophic turbinate and normal turbinate would possess the similar hTMSCs distribution with parallel potency, which meant that there would be the same bony hypertrophy in both turbinates (hypertrophic and normal turbinate) despite NSD only in view of MSCs. However, the expected in-vivo phenomenon contradicts with the known radiologic and histologic findings. Therefore, we assumed that bony turbinate hypertrophy might not result from the characteristics of hTMSCs. However, because this was an *in vitro* study, the possibility of a genetic difference of MSCs stimulating signals in hypertrophic and contralateral turbinates *in vivo* could not be excluded. In addition, further studies at the cellular, biochemical and molecular levels should be performed to permit effective control of hTMSC proliferation and differentiation.

Through this study, because hTMSCs express MSC-specific surface proteins, are highly proliferative, and differentiate into cells with an osteogenic phenotype irrespective of turbinate size, the turbinate size would not be a deciding factor in the clinical use of autologous or allogenic hTMSCs. In addition, these results facilitate a greater understanding of the applicability of hTMSCs to clinical medicine.

## Conclusion

hTMSCs express MSC-specific surface proteins, are highly proliferative, and differentiate into cells with an osteogenic phenotype irrespective of turbinate size. These results suggest that hTMSCs do not contribute to the process of NSD in humans and could facilitate a greater understanding of their characteristics. Therefore, turbinate size would be non-important when deciding whether to use autologous or allogenic hTMSCs. However, further studies such as in vivo tests are required to definitively state the relationship of hTMSCs with turbinate size or the process NSD in humans.
